# Dissimilarity for functional data clustering based on smoothing parameter commutation

**DOI:** 10.1177/0962280217710050

**Published:** 2017-05-24

**Authors:** ShengLi Tzeng, Christian Hennig, Yu-Fen Li, Chien-Ju Lin

**Affiliations:** 1 Department of Public Health, China Medical University, Taiwan; 2 Department of Statistical Science, University College London, UK; 3 MRC Biostatistics Unit, University of Cambridge, UK

**Keywords:** Clustering, irregular longitudinal data, functional data, smoothing splines, dissimilarity, outliers

## Abstract

Many studies measure the same type of information longitudinally on the same subject at multiple time points, and clustering of such functional data has many important applications. We propose a novel and easy method to implement dissimilarity measure for functional data clustering based on smoothing splines and smoothing parameter commutation. This method handles data observed at regular or irregular time points in the same way. We measure the dissimilarity between subjects based on varying curve estimates with pairwise commutation of smoothing parameters. The intuition is that smoothing parameters of smoothing splines reflect the inverse of the signal-to-noise ratios and that when applying an identical smoothing parameter the smoothed curves for two similar subjects are expected to be close. Our method takes into account the estimation uncertainty using smoothing parameter commutation and is not strongly affected by outliers. It can also be used for outlier detection. The effectiveness of our proposal is shown by simulations comparing it to other dissimilarity measures and by a real application to methadone dosage maintenance levels.

## 1 Introduction

Clustering sets out to find groups of subjects based on several different characteristics, where subjects within a cluster are considered to be similar based on the given characteristics. The degree of similarity and dissimilarity can be defined in many ways, and there are many clustering methods, including hierarchical clustering, k-means, DBSCAN, etc. Berkhin^1^ gives an overview of both partition-based and hierarchical clustering methods, Bouveyron and Brunet-Saumard^2^ review popular partition-based methods for high-dimensional data, and Murtagh and Contreras^3^ review several hierarchical clustering algorithms, see also Hennig et al.^4^ A bottom-up hierarchical method does not require any statistical model assumption, rather only a linkage method by which two clusters are merged at each step of the hierarchical agglomerative process. This process can be displayed by a dendrogram from which clusters are obtained. However, once merged, clusters cannot be separated at the next step. Partition-based methods, in contrast, partition subjects into a desired number of clusters, which needs to be specified. The cluster assignment of subjects for one number of clusters for these methods does not restrict cluster assignments for other numbers of clusters.

In many situations, the same type of information on the same subject is measured at multiple time points. To cluster such data, one should take into account the data format and the temporal order structure. Data of each subject are often collected at unequally spaced time points. As a result of aligning records into a conventional ‘variable-by-variable’ format, there are many ‘empty’ records at regular time points. Those empty records can be regarded as a kind of ‘missing’ value. In addition, even if all subjects were observed at the same time points, conventional clustering fails to take into account the temporal order structure of variables, on which adjacent measurements for the same subject are expected to have similar values. Functional data clustering exists for grouping such data. Three major categories for functional data clustering are dissimilarity-based methods, decomposition-based methods and model-based methods.

Dissimilarity-based methods, using pointwise dissimilarities between pairs of subjects, are the most straightforward approach.^5–7^ These methods often take care of data format and time coordinates by certain curve smoothing or imputation techniques, and subsequently dissimilarities between subjects are computed to which the conventional dissimilarity-based methods can be applied. Little attention, however, has been paid to the uncertainty of smoothing or imputation. To the best of our knowledge, the only two exceptions are (a) a prediction-based approach of Alonso et al.^8^ that was modified by Vilar et al.,^9^ and (b) a hypotheses-testing-like approach of Maharaj.^10^ The former is computationally intensive and the latter is designed for invertible ARMA processes, which restricts their application.

Decomposition-based methods overcome the issue of smoothing and sequential order by transforming the observed data into a finite series of common features. These procedures deal with the uncertainty of smoothing implicitly. For example, Abraham et al.^11^ used spline basis functions, James et al.^12^ used functional principal component analysis, and Warren Liao^13^ reviewed more sophisticated ‘feature-extraction’ algorithms. These approaches define common features for all groups and then assign weights to features by which groups are identified. Each group has different weights on those features, and each group can be interpreted according to its lower-dimensional projection on features. Features extracted from certain transformations of data are also popular, such as spectral densities,^14^ periodograms^15,16^ and permutation distributions.^17^ Nonetheless, in reality, not all groups share the same number of features, and it is not easy to determine an appropriate number of dimensions.

In light of the difficulties encountered by the first two methods, many researchers suggest the third alternative: various model-based frameworks. They estimate individual underlying curves and cluster subjects simultaneously, and then statistical inference can be made based on the working models for clusters, such as measuring the uncertainty for cluster assignment and ‘within-cluster’ variation. Unfortunately, these approaches encounter other challenges. Purely parametric functional forms as used in Jones and Nagin^18^ may not be realistic, and its assumption of subjects sharing the same ‘underlying’ curve within a group can be too restrictive. Applying semi- or non-parametric methods usually requires some dimension reduction within each group (e.g. FCM,^19^ funHDDC,^20^ Funclust^21^ and K-centre^22^), which encounters a similar problem as decomposition-based methods. A pure likelihood-based framework (without dimension reduction) called longclust is proposed by McNicholas and Murphy.^23^ This method is limited to short time series and breaks down easily due to the curse of dimensionality. Even worse, the notion of distribution for random functions is not well-defined as curves could have infinite dimensions.^24^

We have reviewed the strengths and weaknesses of the existing functional data clustering methods. Moreover, it is worth mentioning the dilemma resulting from curve variability. Clustering curves can be a difficult ‘chicken-and-egg’ problem between (a) how to determine the within-cluster variations before identifying subgroups, and (b) how to separate subgroups when within-cluster variations are unknown. This dilemma is related directly to the smoothing uncertainty problem in dissimilarity-based approaches. Decomposition- and model-based approaches estimate such variability with necessity, but the magnitude of the estimate is often distorted when outliers occur. A two-step strategy exploiting relative merits of different methods seems reasonable: initially separate potential outliers based on an ‘outlier-invariant’ pairwise dissimilarity, and then form main clusters with another appropriate clustering method. For such a strategy, a dissimilarity measure concerning the variability of curve estimation or feature selection is crucial.

In this article, we develop an easily implementable and practically advantageous dissimilarity measure between subjects. The curve smoothing used here is based on the technique of smoothing splines, which is completely determined by the chosen smoothing parameter. With an infinite smoothing parameter, the curve is estimated as a straight line, while the curve interpolates the observed data with a zero smoothing parameter. The innovation of our method is to measure the dissimilarity between subjects based on pair-by-pair varying curve estimates for a subject. The concepts are that (a) smoothing parameters of smoothing splines reflect the inverse of the signal-to-noise ratios and (b) the estimated curves for two similar subjects are expected to be close if an identical smoothing parameter is applied to both sets of observations. Specifically, if the unobserved true curves of subjects *i* and *j* are similar, their curve estimates should resemble with each other, no matter whether we use a smoothing parameter primarily for the *i*-th or the *j*-th subject. Our dissimilarity is then calculated through commuting between the smoothing parameters for a pair.

The rest of the article is organized as follows. Section 2 describes the proposed Smoothing Parameter Commutation dissimilarity and some of its properties. Its effectiveness is shown through simulations comparing to other dissimilarity measures in Section 3. An example of its application to methadone dosages observations is given in Section 4, where we also identify outliers with a simple but efficient method. Finally, Section 5 provides some concluding remarks and discussion concerning future directions.

## 2 Method

### 2.1 Smoothing splines

We utilize the smoothing spline to estimate curves of subjects. Assume that the curve of the *i*-th subject is observed as a set of measurements $\left\{y_{i,1},\ldots ,y_{i,K_{i}}\right\}$ contaminated by noises at distinct finite time points $\left\{t_{i,1},\ldots ,t_{i,K_{i}}\right\}$ in an interval $\left[T_{L},T_{U}\right]$ according to the model

(1) $$y_{i,k}=f_{i}(t_{i,k})+\varepsilon_{i,k},k=1,\ldots ,K_{i},i=1,\ldots ,n$$

where $f_{i}(\cdot )$ is the function of the true curve, and at time $t_{i,k}$ the noise $\varepsilon_{i,k}∼{\;}^{\mathrm{iid}}N(0,\sigma^{2})$ and the true value $f_{i}(t_{i,k})$ are both unobservable. A reasonable estimation of *f_i_* is to minimize $\frac{1}{K_{i}}\sum_{k}(y_{i,k}-f_{i}(t_{i,k})){\;}^{2}$ while controlling the smoothness of *f_i_* by requiring ∫TLTU(fi''(t)) 2dt≤ρ for a positive *ρ*. This estimator is equivalent to a smoothing spline fi^(·;λ) that minimizes

(2) 1Ki(yi-fi)'(yi-fi)+λ∫TLTU(fi''(t)) 2dt

where $y_{i}=(y_{i,1},\ldots ,y_{i,K_{i}})'$ and $f_{i}=(f_{i}(t_{i,1}),\ldots ,f_{i}(t_{i,K_{i}}))'$ given a smoothing parameter λ≥0.^25,26^ With an infinite *λ* the curve fi^ is a straight line, while with *λ* = 0, fi^ interpolates exactly all the data points. There are various methods to determine an appropriate *λ* in (2), and once it is chosen, f^i(t;λ) is completely determined over $t\in [T_{L},T_{U}]$. We exploit a mixed-effects model representation^27^ to choose *λ* in (2), which formulates $y_{i}$ as

(3) $$y_{i}=X_{i}\beta_{i}+u_{i}+\varepsilon_{i}$$

where $\beta_{i}$ is the fixed effect, $X_{i}$ has two columns being 1’s and $(t_{i,1},\ldots ,t_{i,K_{i}})'$, $\varepsilon_{i}=(\varepsilon_{i,1},\ldots ,\varepsilon_{i,K_{i}})'∼N(0,\sigma^{2}I)$, and $u_{i}∼N(0,\sigma_{u}^{2}\Psi )$ with $\sigma_{u}^{2}=\sigma^{2}/(K_{i}\lambda )$ and a specific correlation Ψ. Speed^28^ pointed out that minimizing (2) is equivalent to finding the minimum variance linear unbiased predictor of $y_{i}$ in (3) with *λ* fixed. They dealt with coordinates based on transformed values. For $t,\tau \in [T_{L},T_{U}]$, let $\overset{∼}{t}=(t-T_{L})/(T_{U}-T_{L})$ and $\overset{∼}{\tau }=(\tau -T_{L})/(T_{U}-T_{L})$, then $\overset{∼}{t},\overset{∼}{\tau }\in [0,1]$. They use a correlation matrix with its $\left(k,k^{*}\right)$-th element being $\int_{0}^{1}({\overset{∼}{t}}_{i,k}-\overset{∼}{\tau }){\;}_{+}({\overset{∼}{t}}_{i,k^{*}}-\overset{∼}{\tau }){\;}_{+}\text{d}\overset{∼}{\tau }$, where $a_{+}=\max(0,a)$. That is, setting the $\left(k,k^{*}\right)$-th element of Ψ to be a function of $t_{i,k}$ and $t_{i,k^{*}}$ as

(4) $$\begin{array}{l} \int_{T_{L}}^{T_{U}}\frac{{\left\{(t_{i,k}-T_{L})-(\tau -T_{L})\right\}}_{+}}{T_{U}-T_{L}}\frac{{\left\{(t_{i,k^{*}}-T_{L})-(\tau -T_{L})\right\}}_{+}}{T_{U}-T_{L}}(T_{U}-T_{L})-1d\tau \\ ={\left(T_{U}-T_{L}\right)}^{-3}\int_{T_{L}}^{T_{U}}{\left(t_{i,k}-\tau \right)}_{+}(t_{i,k^{*}}-\tau )+d\tau . \end{array}$$

For any given $t_{i,k}$ and $t_{i,k^{*}}$ in (4), $(t_{i,k}-\tau ){\;}_{+}$ and $(t_{i,k^{*}}-\tau ){\;}_{+}$ are two truncated linear functions over $\tau \in [T_{L},T_{U}]$, and the integral is a convolution of these two functions, which does not depend on *τ*. Note that if one does not treat *λ* as fixed, *λ* can be expressed as a function of the variance of $u_{i}$ in (3). Under the Gaussian assumption for $\varepsilon_{i}$ and $u_{i}$, the two variance components $\sigma_{u}^{2}$ and $\sigma^{2}$ can be determined based on the restricted maximum likelihood method (REML), so that *λ* is also determined, and $K_{i}\lambda$ has a useful interpretation as the inverse of the signal-to-noise ratio $\sigma_{u}^{2}/\sigma^{2}$. Additionally, it has been shown that the smoothing results based on (3), (4) and REML are robust even when the correlation structure of $\varepsilon_{i}$ is mis-specified.^27,29^

### 2.2 Smoothing Parameter Commutation dissimilarity

The concept of our method is that if the ‘true’ *f_i_* and *f_j_* are similar, it is expected that f^i and f^j from $y_{i}$ and $y_{j}$ should be close, given an identical smoothing parameter. Our proposal starts with finding λ^i in (3) for a subject *i* based on $y_{i}$. The estimated curve is denoted by f^i(·;λ^i), which means that f^i(·;λ) is estimated by setting λ=λ^i in (2) based on the observations $y_{i}$. Given λ^i, we can also obtain f^j(·;λ^i) based on the observations $y_{j}$. Similarly, we exchange the roles of the two subjects to obtain f^j(·;λ^j) and f^i(·;λ^j). The dissimilarity between subjects *i* and *j* is defined as

(5) di,j=12{[∫TLTU(f^i(t;λ^i)-f^j(t;λ^i))2dt]1/2+[∫TLTU(f^i(t;λ^j)-f^j(t;λ^j))2dt]1/2}

Due to the roles of λ^i and λ^j in (5), we call it a Smoothing Parameter Commutation dissimilarity. It takes the variation of smoothing into consideration with different *λ*’s for different pairs of (*i*, *j*)’s, rather than focusing on the dissimilarity between (fixed) estimated curves. Note that $d_{i,j}\geq 0$ and $d_{i,j}=0$ if *i* = *j*, and it is clear that $d_{i,j}=d_{j,i}$, so conventional dissimilarity-based clustering methods can be applied. Note that the triangle inequality cannot be proved, in general, hence the term ‘dissimilarity’ rather than ‘distance’, but this is not required for dissimilarity-based clustering methods. The dissimilarity reduces to rooted integral squared difference of *f_i_* and *f_j_* when no missing values and measurement errors are present.

Our proposal has several advantages. First, data observed at irregular time points can be handled directly, because of the nature of smoothing splines. Second, the dissimilarity also serves as a useful tool for outlier detection (see Section 4). Third, the implementation is handy since subroutines for smoothing splines and numerical integration are widely available. Although the computational burden for (5) seems heavy at first glance, it can be done quite efficiently among *n* subjects. Given *λ*, a fast $O(K_{i})$ algorithm to compute fi^(t;λ) does exist.^30^ Thus, one needs to solve λ^i in (3) only *n* times for the *n* subjects, and then one adopts the fast algorithm for {f^j(t;λ^i):i,j=1,…,n}. Therefore, the computational complexity is proportional to that in treating f=fi^(t;λi) as fixed and calculating the dissimilarity as square root of ∫TLTU(f^i(t;λ^i)-f^j(t;λ^j))2dt (the latter procedure is referred to as *d_SS_* in what follows).^31^

## 3 Simulation study

We conduct a simulation to investigate whether our proposed Smoothing Parameter Commutation measure is more capable than other dissimilarity measures when observations are contaminated with (independent or dependent) noises. If an analyst is interested in the relative shape patterns of curves, regardless of shift, shrinkage, expansion or magnitude, then several alignment, normalization and warping tools can be applied in preprocessing.^32–34^ In order to not lose focus, we do not consider dissimilarity measures engaging with the preprocessing.

We consider the following four random curve models over t∈[0,1]:

$$\begin{array}{l} f^{\left(1\right)}(t;\eta )=3\eta ,\\ f^{\left(2\right)}(t;\eta )=\sin(2\pi t)-t+2\eta \cos(4\pi t),\\ f^{\left(3\right)}(t;\eta )=3t+2\eta t,\\ f^{\left(4\right)}(t;\eta )=5\eta \left\{{\left(t-0.5\right)}^{2}-2t(1-t)\right\}, \end{array}$$

where $\eta ∼N(1,0.3^{2})$. The four functional forms represent constant, periodic, linear and nonlinear (unobserved) true curves, respectively. The observed data are generated according to (1) at 200 time points, $t_{k}\in \{0,1/199,\ldots ,198/199,1\}$, with noise coming from four mechanisms

(6) $$\begin{array}{l} \text{WN}:\;\;\;\epsilon_{k}=\xi_{k},\\ \text{AR}:\;\;\;\;\epsilon_{k}=0.8\epsilon_{k-1}+\xi_{k},\\ \text{SARMA}:\;\;\epsilon_{k}=0.8\epsilon_{k-10}+0.8\xi_{k-10}+\xi_{k},\\ \text{BILR}:\;\;\;\epsilon_{k}=0.8\epsilon_{k-1}+0.2\xi_{k-1}-0.2\epsilon_{k-1}\xi_{k-1}+\xi_{k}, \end{array}$$

where $\xi_{k}∼{\;}^{\mathrm{iid}}N(0,1)$, and *ξ_k_* is independent of $\varepsilon_{k'}$ for k'≠k. That is, we set $K_{i}\equiv 200,t_{\mathrm{ik}}\equiv (k-1)/199$. The four noise mechanisms are examples of usual assumption for noises: a purely independent process, a stationary process, a cyclostationary process and a non-stationary process. For each combination of $f\in \{f^{\left(1\right)},f^{\left(2\right)},f^{\left(3\right)},f^{\left(4\right)}\}$ and mechanism *ε_k_*, 10 series are generated according to 10 independent *η*’s as well as 10 sets of *ε_k_*’s. In total, there are 160 series mimicking the longitudinal observations from 160 subjects.

Ten dissimilarity measures as listed in Table 1 are applied to the simulated data. They include seven measures in Montero and Vilar,^35^ the proposed Smoothing Parameter Commutation method (*d_SPC_*), point-wise Euclidean distance (*d_EUCL_*), and *d_ss_* as mentioned in the last section. Two comparison criteria are defined as follows:

$$\begin{array}{l} Q=n^{-2}\underset{a,b}{\min}\sum_{i=1}^{n}\sum_{j\neq i}^{n}\frac{{\left(a+b{\hat{d}}_{i,j}-d_{i,j}\right)}^{2}}{d_{i,j}},\\ R=n^{-2}\sum_{i=1}^{n}\sum_{j\neq i}^{n}{\left({\hat{r}}_{i,j}-r_{i,j}\right)}^{2}, \end{array}$$

where d^i,j is one of the considered dissimilarity measures between the *i*-th and *j*-th subjects, $d_{i,j}=\sqrt{\sum_{k}(f_{i}(t_{\mathrm{ik}})-f_{j}(t_{\mathrm{jk}})){\;}^{2}}$ is the true dissimilarity without noise, and r^i,j and $r_{i,j}$ are the corresponding ranks of d^i,j and $d_{i,j}$ among all pairs of (*i*, *j*)’s, respectively. The quantity *Q* reflects the loss, normalized by the true dissimilarity scales, for (linear) approximation to all the pairs of true dissimilarities, while *R* measures the deviation from monotonicity between d^i,j and $d_{i,j}$. A good measure should have a small value of *Q* and *R*. The averaged *Q*- and *R*-values for the 10 measures over 200 simulation replicates are given in Tables 2 and 3, respectively.

**Table 1.** Dissimilarity measures to be compared.

Notation	Description	Literature
*d_EUCL_*	Point-wise Euclidean distance	$\sqrt{\sum_{k}(y_{\mathrm{ik}}-y_{\mathrm{jk}}){\;}^{2}}$
*d_SPC_*	Smoothing Parameter Commutation	The proposed method
*d_MAH_*	Parametric testing of equality of processes	Maharaj^10^
*d_GLK_*	Nonparametric equality testing of log-spectra	Fan and Zhang^14^
*d_SS_*	Based on spline smoothing curves	Ramsay and Silverman^31^
*d_CORT_*	Correlation-based modification of *d_EUCL_*	Chouakriya and Nagabhushan^36^
*d_IP_*	Based on integrated periodogram	de Lucas^16^
$d_{\mathrm{PRED},h}$	Based on predicted values at future	Vilar et al.^9^
*d_CID_*	Complexity-based modification of *d_EUCL_*	Batista et al.^37^
*d_PDC_*	Permutation distributions of order patterns	Brandmaier^17^

**Table 2.** Averaged *Q*-values over 200 simulated replicates among 10 dissimilarity measures for each combination of *f* and *ε_k_* (with 10 random curves), and all the 160 curves.

	*d_EUCL_*	*d_SPC_*	*d_MAH_*	*d_GLK_*	*d_SS_*	*d_CORT_*	*d_IP_*	$d_{\mathrm{PRED},h}$	*d_CID_*	*d_PDC_*
$f^{\left(1\right)}$ + W	**1.59**	**0.36**	8.45	8.55	**0.37**	3.82	9.32	1.85	2.19	8.63
$f^{\left(1\right)}$ + A	**5.61**	**5.66**	8.07	8.02	**5.66**	6.4	9.54	**4.61**	5.82	7.96
$f^{\left(1\right)}$ + S	8.07	**6.15**	8.67	8.55	**6.16**	8.68	10.53	**7.32**	8.19	8.78
$f^{\left(1\right)}$ + B	**7.65**	**7.66**	8.48	8.48	**7.65**	7.89	11.88	**5.62**	7.87	8.48
$f^{\left(2\right)}$ + W	2.21	**0.87**	3.79	3.56	**0.83**	3.54	**1.35**	3.96	2.76	4.06
$f^{\left(2\right)}$ + A	**3.94**	**3.94**	**3.91**	3.97	**3.94**	**3.94**	5.69	4.01	**3.95**	3.97
$f^{\left(2\right)}$ + S	3.96	**3.81**	3.83	**3.83**	**3.63**	3.94	5.71	4.04	3.93	3.95
$f^{\left(2\right)}$ + B	**3.99**	**3.99**	4.05	4.06	**3.99**	**3.99**	12.32	4.04	4.04	4.09
$f^{\left(3\right)}$ + W	1.49	**1.05**	1.40	1.40	**0.99**	1.52	**1.38**	1.58	1.50	1.53
$f^{\left(3\right)}$ + A	**1.49**	**1.49**	**1.47**	**1.49**	**1.49**	1.50	2.38	1.51	**1.49**	**1.49**
$f^{\left(3\right)}$ + S	1.53	**1.49**	**1.49**	**1.49**	**1.49**	1.52	4.07	1.54	1.52	1.51
$f^{\left(3\right)}$ + B	**1.56**	**1.56**	**1.55**	**1.56**	**1.56**	1.57	12.44	1.58	**1.56**	**1.56**
$f^{\left(4\right)}$ + W	**2.31**	**0.79**	3.17	3.19	**0.81**	2.94	3.53	2.69	2.54	3.18
$f^{\left(4\right)}$ + A	**3.20**	**3.20**	3.21	3.27	**3.20**	3.23	4.01	**3.04**	3.23	3.22
$f^{\left(4\right)}$ + S	3.29	**3.19**	3.23	**3.22**	**3.18**	3.29	4.74	3.32	3.28	3.25
$f^{\left(4\right)}$ + B	**3.35**	**3.35**	3.38	3.38	**3.35**	3.36	9.01	3.31	3.38	3.37
ALL	**24.83**	**23.92**	29.29	29.30	**24.45**	26.13	32.38	25.63	28.86	29.28

W, A, S and B in the first column stand for WN, AR, SARMA and BILR in (6), respectively. Bold digits are the best 3 within each row.

**Table 3.** Averaged *R*-values over 200 simulated replicates among 10 dissimilarity measures for each combination of *f* and *ε_k_* (with 10 random curves), and all the 160 curves.

	*d_EUCL_*	*d_SPC_*	*d_MAH_*	*d_GLK_*	*d_SS_*	*d_CORT_*	*d_IP_*	$d_{\mathrm{PRED},h}$	*d_CID_*	*d_PDC_*
$f^{\left(1\right)}$ + W	**0.73**	**0.24**	12.26	12.15	**0.24**	2.22	12.01	1.11	1.16	12.39
$f^{\left(1\right)}$ + A	**4.89**	**4.89**	12.11	12.02	**4.89**	5.85	12.29	**3.98**	5.15	12.29
$f^{\left(1\right)}$ + S	7.79	**5.21**	11.82	11.94	**5.24**	10.20	12.29	**7.23**	8.87	12.18
$f^{\left(1\right)}$ + B	**7.73**	**7.73**	12.27	12.15	**7.73**	8.30	12.25	**4.70**	8.20	12.43
$f^{\left(2\right)}$ + W	3.01	**1.04**	8.88	6.69	**1.01**	6.27	**1.29**	11.69	4.21	12.27
$f^{\left(2\right)}$ + A	**9.20**	**9.20**	10.24	10.59	**9.19**	9.80	8.15	12.66	9.45	12.05
$f^{\left(2\right)}$ + S	10.99	**8.19**	10.14	10.18	**7.88**	11.71	**7.85**	13.45	11.33	12.35
$f^{\left(2\right)}$ + B	**10.59**	**10.6**	11.62	11.63	**10.6**	10.89	10.77	12.66	10.75	12.10
$f^{\left(3\right)}$ + W	9.18	**4.49**	8.16	8.10	**4.27**	10.79	**6.78**	14.54	10.09	12.35
$f^{\left(3\right)}$ + A	**11.5**	**11.53**	11.89	11.87	**11.53**	11.69	12.06	13.88	**11.53**	12.22
$f^{\left(3\right)}$ + S	11.90	**11.53**	**11.72**	12.17	**11.34**	11.95	12.10	14.09	11.87	11.96
$f^{\left(3\right)}$ + B	**11.99**	**12.02**	12.12	12.05	**12.02**	**11.94**	12.26	13.51	12.06	12.31
$f^{\left(4\right)}$ + W	**4.63**	**1.31**	11.56	12.23	**1.32**	7.87	12.29	7.49	5.79	12.21
$f^{\left(4\right)}$ + A	**9.89**	**9.89**	11.59	12.28	**9.88**	10.45	12.47	10.00	10.02	12.18
$f^{\left(4\right)}$ + S	11.71	**10.23**	**11.34**	12.11	**10.23**	11.87	12.19	13.08	11.72	12.20
$f^{\left(4\right)}$ + B	**11.24**	**11.26**	12.24	12.20	**11.26**	11.52	12.24	**10.83**	11.41	11.68
ALL	**1155.6**	**874.7**	4160.5	4063.7	**901.0**	1315.4	3768.6	1239.8	2693.1	4191.6

W, A, S and B in the first column stand for WN, AR, SARMA and BILR in (6), respectively. Bold digits are the best 3 within each row.

The *Q* value is a loss function based on the best affine approximation, which measures how close optimally linearly transformed dissimilarity measures are to the true *d_ij_*. Obviously, this would be a good feature for a dissimilarity measure in such cases, but one may argue that achieving this is not the aim of some of the measures listed in Table 1, so we propose alternative measures, see below. *Q* favours Euclidean distance based dissimilarities. R measures by and large the same feature but does not rely on metric approximation. Note that in areas of high density of curves small changes in the metric approximation quality can lead to much larger changes in ranks, so that differences in *R* are often much clearer than differences in *Q*. In any case, the two comparison criteria are highly coherent in that they almost always detect the same best and worst measures. As expected, without missing data, *d_EUCL_* is often among the best measures and it looks unbiased for estimating the true distance between curves in many situations. But it does not do well enough if the signal or noise is periodic ($f^{\left(2\right)}$, SARMA, respectively). Our *d_SPC_* method and *d_SS_* always are among the best three measures, both for the 10 curves within an individual group and for 160 curves as a whole. The non-stationarity of BILR can occasionally lead to larger-variance noises. As a result, noises mask the signal at some time points. When the signal-to-noise ratio becomes lower, most dissimilarity measures cannot perform well. As expected, if noises come from BILR, *d_SPC_* and *d_SS_* have a smaller advantage, that is, their performances are not very different from other methods. Note that *d_SS_* and *d_SPC_* yield an almost identical result within groups, due to them both utilizing the mixed-effects model representation of smoothing splines. The difference lies in that *d_SS_* regards f^i(t;λ^i) as a fixed estimate of *f_i_*. Our *d_SPC_* method outperforms the others for between-group dissimilarity, which indicates the advantage of accounting for smoothing variation via smoothing parameter commutation. In certain cases $d_{\mathrm{PRED},h}$ and *d_MAH_* are good measures, which also take estimation uncertainty into consideration.

For cases where the heterogeneities in magnitude and in shape are of interest, criteria targeting at the integrated euclidean distances are reasonable to use as a comparison index, whereas the criteria might not be sensible in applications such as segregating relative shape changes regardless of magnitudes in microarray experiments.^38^ Therefore, in addition to *Q* and *R*, we examined the cluster recovery ability among measures list in Table 1 with the Nowak index, Rand index and adjusted Rand index. The Nowak index focuses on the largest cluster, the Rand index measures the average performance, and the adjusted Rand index is a chance-corrected version of the Rand index. All are implemented in Dudek^39^ and the reference therein gives the detailed definitions. There are four signal patterns in our simulation setup, despite various noise mechanisms. We applied ‘partitioning around medoids’ clustering (PAM^40^) with four clusters to the pairwise dissimilarity matrix of each measure. The true clusters were defined by the four random curve models. The average values and standard errors of indices for the 10 measures over 200 simulation replicates are given in Table 4. *d_SPC_*, *d_SS_*, *d_CORT_* and *d_EUCL_* outperform the other measures, and *d_SPC_* is the best among the four by a small margin.

**Table 4.** Averaged measures over 200 simulated replicates among 10 dissimilarity measures for four groups of *f* among all the 160 curves.

	*d_EUCL_*	*d_SPC_*	*d_MAH_*	*d_GLK_*	*d_SS_*	*d_CORT_*	*d_IP_*	$d_{\mathrm{PRED},h}$	*d_CID_*	*d_PDC_*
RAND	0.7705	0.8034	0.6179	0.6174	0.8016	0.772	0.6156	0.7295	0.7015	0.6139
	(0.0038)	(0.0015)	(0.0025)	(0.0026)	(0.0013)	(0.0039)	(0.0027)	(0.0034)	(0.00329)	(0.00256)
adjRAND	0.4154	0.4998	−0.0156	−0.014	0.4962	0.4205	0.0269	0.3129	0.2332	−0.0062
	(0.0043)	(0.0034)	(0.0002)	(0.0003)	(0.0027)	(0.0046)	(0.0009)	(0.0029)	(0.00415)	(0.00044)
Nowak	0.5679	0.6181	0.2607	0.2654	0.6165	0.573	0.3079	0.467	0.428	0.2786
	(0.0042)	(0.0040)	(0.0011)	(0.0012)	(0.0035)	(0.0046)	(0.0019)	(0.0027)	(0.0022)	(0.0016)

Digits in parentheses are the standard errors.

## 4 Real data application with outlier detection

We apply *d_SPC_* defined in (5) to a methadone maintenance therapy dataset analysed in Lin et al.^41^ Daily methadone dosages in milligrams (mg) for 314 participants between 01 January 2007 and 31 December 2008 were collected. Dosage records for each patient from day 1 to 180 were used for clustering. Lin et al.^41^ categorized the dosages into seven levels, one of which is for missing values, and proposed a dissimilarity measure for clustering such ordinal data with extra missingness category. The ordering of time coordinates, however, was ignored in their approach. In this example, we use the daily dosage taken by patients, and do not recode missing values separately. Smoothing splines take care of the irregular follow-up time points of patients automatically, which may not be an easy task for some other measures listed in Table 1.

Real data, inevitably, are prone to have outliers. Garcia-Escudero et al.^42^ give an overview of the impact of outliers on clustering and some approaches to deal with them. A clustering procedure with outlier removal consists of three steps: (a) calculating the dissimilarity matrix, (b) detecting and removing outliers and (c) grouping the remaining subjects into a desired number of clusters. An outlying curve in functional data is not only one with a few unusual high or low measurements, but also one that has an overall atypical magnitude or shape. Hubert et al.^43^ distinguish the former as ‘isolated outlier’ and the latter as ‘persistent outlier’. Among ‘persistent outliers’, Hyndman and Shang^44^ call curves lying outside the range of the vast majority of the data ‘magnitude outliers’ and call those having a very different shape from other curves ‘shape outliers’. Much research has been done on detection of persistent outliers.

Some work exploits the notion of data depth for sorting subjects into layers with a more outward layer more likely to be atypical (first proposed by Tukey^45^; see Gervini^46^ and Hubert et al.^43^ for an overview in the functional setting), often equipped with a functional boxplot as a visualization tool. Some methods rely on robust functional principal components,^47,48^ which brings about various visualization tools such as bagplots and highest-density-region boxplots.^44^ While most of the above focus on magnitude outliers, Arribas-Gil and Romo^49^ propose the ‘outliergram’ to detect shape outliers as well as magnitude outliers. Other methods deal with phase heterogeneity relating to warping and alignment preprocessing,^50,51^ which is beyond the scope of this work.

The aforementioned methods regard outliers as subjects that are very different to the majority. Alternatively, Ramaswamy et al.^52^ propose a dissimilarity-based outlier detection method considering the dissimilarity to nearest neighbours. Their outlier definition is that outliers are those with no or only so few subjects nearby that these could not be interpreted as forming a relevant cluster. This method has been shown to be effective in the pattern recognition literature.^53,54^ The difficulty in applying it to functional data is the construction of an appropriate dissimilarity, which is the main theme of the present study. By virtue of the good performance of *d_SPC_* in the previous section, we consider a dissimilarity-based outlier detection method similar to Ramaswamy et al.^52^ We obtain a pairwise dissimilarity matrix based on (5), and calculate the average dissimilarity of each participant to their *k* nearest neighbours. The minimum size of clusters to be meaningful (*k* should be this size minus one) in principle depends on the application and the size of the dataset. Averaging the dissimilarities to the nearest neighbours implies that a collection of up to *k* subjects needs to be further away from the remaining subjects in order to be considered outliers than a single isolated subjects, because a single isolated outlier forms an even less relevant pattern. Note that in cluster analysis with outliers, there is an essential ambiguity about whether a small group of atypical subjects is a cluster on its own, or rather a group of outliers, see Garcia-Escudero et al.^42^ Therefore, outlier detection methods in clustering will always require some tuning of this kind.

**Figure 1.**
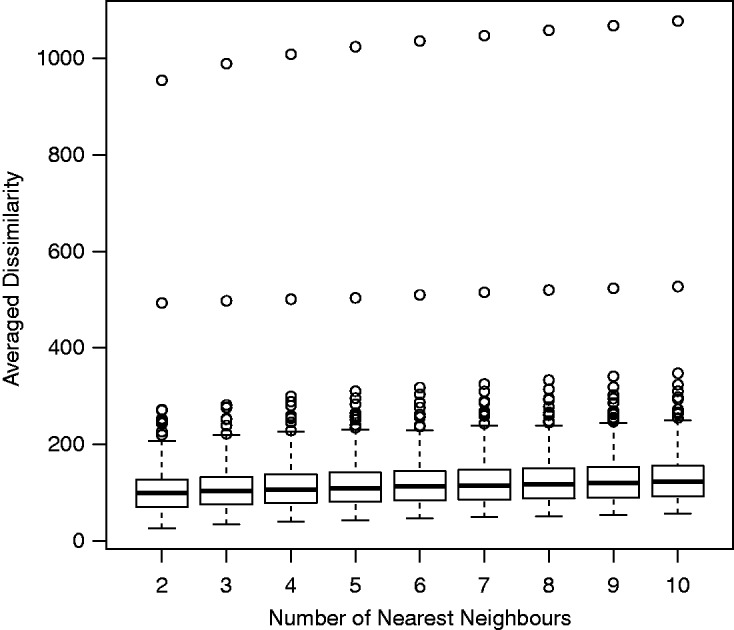
Distribution of the averaged dissimilarity *d_SPC_* to the nearest *k* neighbours, where k=2,…,10.

Two participants had average *d_SPC_* values to the three nearest neighbours as 498 and 989, while the others had values between 34 and 282. The two participants were, therefore, considered as potential outliers. We assess the outlyingness of observations visually, using boxplots, without the need of involving formal model assumptions. Figure 1 shows the average dissimilarities to *k* nearest neighbours, where k=2,…,10. As is clear from the graph, the two participants with the largest nearest neighbour dissimilarities are very far away from the other patients, regardless of *k*.

**Figure 2.**
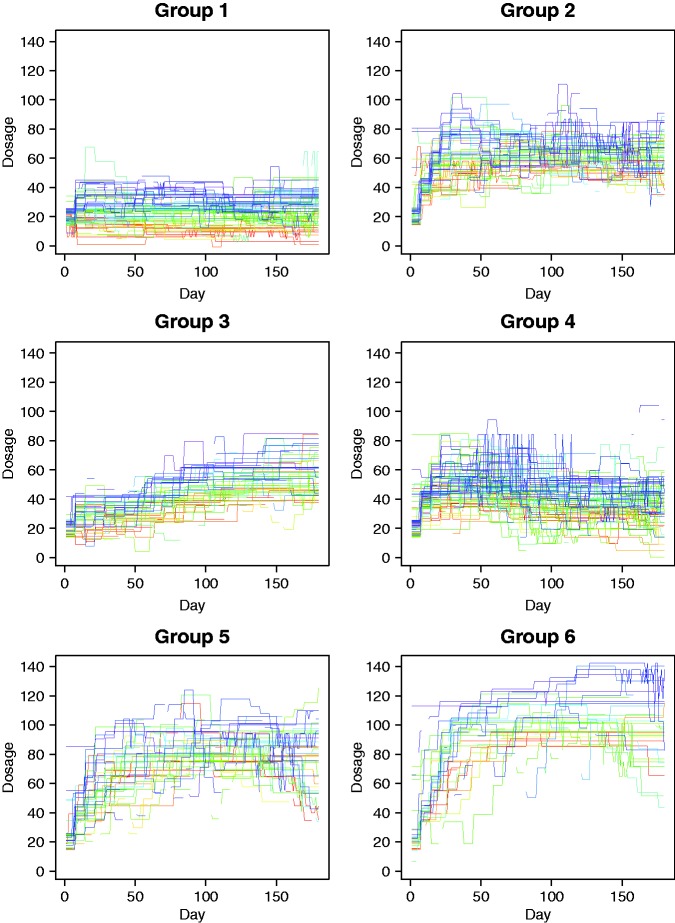
Subgroups from PAM clustering of the 312 patients in methadone maintenance therapy.

**Figure 3.**
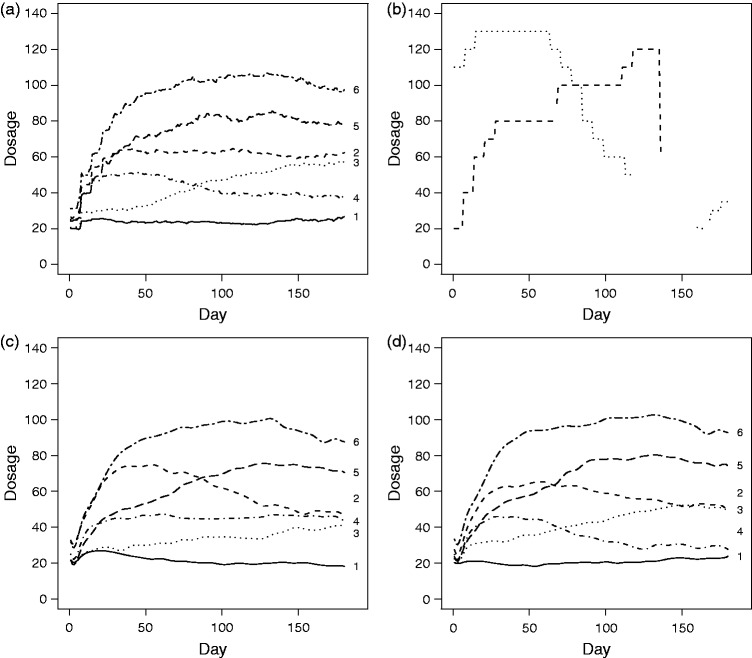
(a): Curves of average dosage of the six groups obtained from PAM and the pairwise dissimilarity matrix in Figure 2; (b) dosage profiles of the two potential outliers; (c) mean profiles of a model-based clustering method including outliers; (d) mean profiles of the model-based clustering method excluding outliers.

Hennig and Lin^55^ used a flexible parametric bootstrap method to assess the number of clusters for the data in Lin et al.^41^ The PAM solutions between 2 and 20 clusters were compared regarding the average silhouette width^40^ and the prediction strength.^56^ This suggested either five or six clusters. We use PAM with six clusters and use the adjusted Rand index to measure the similarity between clusters found in our study. We excluded the two outlying participants and applied PAM with six clusters to the pairwise *d_SPC_* matrix of the 312 participants. Figure 2 shows the clustering result. Each horizontal line represents a dosage curves from day 1 to 180. Figure 3(a) shows the average dosage of each cluster. As seen, Groups 1 and 2 are more stable, remaining at dosages in [10,40] and [40,80], respectively. Group 3 has an upward trend while Group 4 has a downward trend, and their average dosages represented by the two curves cross around day 85. Group 5 goes up quickly and stays at dosage of around 80 mg. Although Group 6 has a similar trend to Group 5, it fluctuates heavily over a larger range and looks less stable. Overall, these figures indicate that a patient with early higher dosage taken (roughly above 60 mg before day 45) does not tend to reduce the level afterwards, and a monitoring between the second and third month can be critical.

Results based on \a model-based functional data clustering are also given for reference. We used the ‘funcit’ function in the ‘funcy’ package^57^ on the Comprehensive R Archive Network (CRAN; R Core Team^58^). The model option of ‘funcit’ is set to be ‘iterSubspace’, i.e. an implementation of the algorithm in Chiou and Li.^22^

The ‘funcy’ package integrates several model-based clustering methods for functional data, but most of them require regular measurements and do not fit the methadone dosage example with many missing values. The only two methods of the package allowing irregular measurements are ‘fitfclust’ and ‘iterSubspace’, and we used the latter because of the length of time and amount of memory needed to run one iteration.

Profiles of the two aforementioned potential outliers are shown in Figure 3(b). The theoretical mean profiles of clusters obtained from the model-based clustering with and without outliers are shown in Figure 3(c) and (d), respectively. Excluding the two outliers improved the model-based method in the sense that the average dissimilarity to the mean profile was reduced by 7.6% from 166.7 to 154.9, which yielded more compact clusters. It seems inappropriate to assign the two outlying curves into the found groups. Enforcing their inclusion will exaggerate the within-group variation, no matter which groups they are assigned to. Then the boundaries of groups are more blurred, and the mean profiles are less representative of their groups.

**Figure 4.**
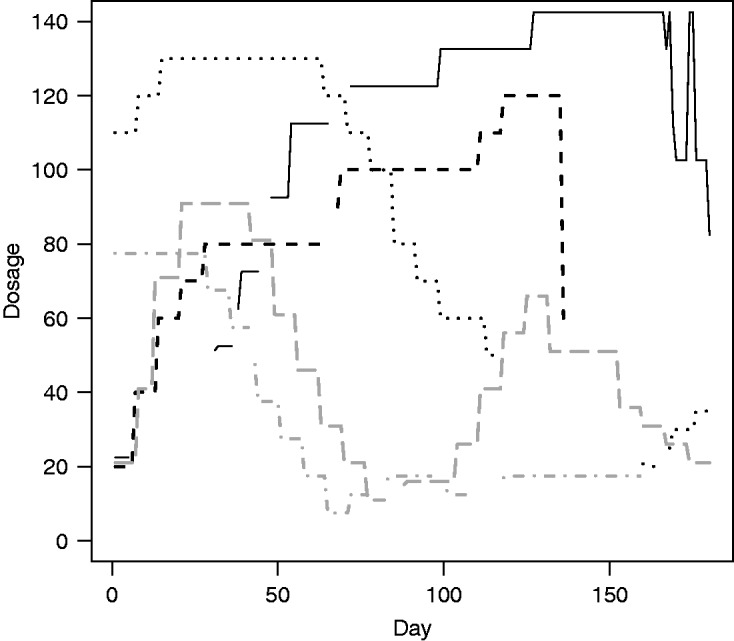
Dosage profiles of the five potential outliers identified by using outligram.

We also applied the outliergram^49^ for outlier detection. Five outliers were detected. Two of them were the same participants as identified by our method shown in Figure 3(b), confirming their outlyingness. The two grey curves in Figure 4 were clustered as Group 4 in Figure 2 but are detected as outlier by the outliergram because of an atypical downward trend, and the black solid curve was in Group 6. For applications where both magnitude and shape heterogeneities are important, our method taking into account both is preferred. In situations where separation is important, the outliergram may be more suitable.

Identifying outliers in model-based clustering is hard, because the assessment of outlyingness depends on the model fit, which in turn is based on the outlying subjects (see Garcia-Escudero et al.^42^ for various iterative approaches for doing this, which depends on some initialization). In contrast, in dissimilarity-based clustering, outliers can be identified based on the dissimilarities alone, without having to rely on knowing the clusters. The simulations above show a strong and stable performance of the proposed dissimilarity. It can also be used for a beneficial pre-cleaning step for model-based clustering.

## 5 Conclusion and discussion

We have shown that the proposed Smoothing Parameter Commutation dissimilarity is good at reproducing the true distances between noiseless curves that change gradually. On the real dataset, dissimilarity-based clustering on smoothed data is superior to model-based clustering under specific time series assumptions. The concept of the proposed dissimilarity measure is simple and easy to implement. We also demonstrated a simple method for outlier detection that helps model-based functional data clustering to form more compact subgroups.

There are many nonparametric regression methods other than smoothing splines, e.g. local polynomial regressions and wavelet analysis. Different techniques stand out in different situations. It is of interest to study whether there exist analogous parameter commutation operations and with similar advantages when applying other nonparametric regressions. This direction is left as a future work.
